# Safety and Efficacy Assessment of Isoflavones from *Pueraria* (Kudzu) Flower Extract in Ovariectomised Mice: A Comparison with Soy Isoflavones

**DOI:** 10.3390/ijms20122867

**Published:** 2019-06-12

**Authors:** Yuko Tousen, Jun Takebayashi, Takashi Kondo, Hiroyuki Fuchino, Noriaki Kawano, Takayuki Inui, Kayo Yoshimatsu, Nobuo Kawahara, Yoshiko Ishimi

**Affiliations:** 1 National Institute of Health and Nutrition, National Institutes of Biomedical Innovation, Health and Nutrition, 1-23-1 Toyama, Shinjuku-ku, Tokyo 162-8636, Japan; tousen@nibiohn.go.jp (Y.T.); jtake@nibiohn.go.jp (J.T.); tkondo@nibiohn.go.jp (T.K.); 2 Research Center for Medicinal Plant Resources, National Institutes of Biomedical Innovation, Health and Nutrition,1-2 Hachimandai Tsukuba-shi, Ibaraki 305-0843, Japan; fuchino@nibiohn.go.jp (H.F.); nkawano@nibiohn.go.jp (N.K.); inui@nibiohn.go.jp (T.I.); yoshimat@nibiohn.go.jp (K.Y.); kawahara@nibiohn.go.jp (N.K.); 3 Tokyo University of Agriculture, NODAI Research Institute, 1-1-1, Sakuragaoka, Setagaya-ku, Tokyo 156-8502, Japan

**Keywords:** isoflavones, *Pueraria* flower, oestrogenic effects, CYP, Cytochrome P-450, kudzu, tectorigenin

## Abstract

Numerous Foods with Function Claims that contain the extract of *Pueraria* flower (kudzu) isoflavones (PFI) are available in the Japanese market. These are labelled with function claims of reducing visceral fat. However, these foods have not undergone proper safety assessment such as the evaluation of their oestrogenic activity and effects on drug-metabolising enzymes (cytochrome P-450: CYP) in the liver. This study evaluated the estrogenic effect and the hepatic CYP activity and mRNA expression in normal female mice as a safety assessment of PFI (Experiment 1). In addition, the bone mineral density and visceral fat weight in ovariectomised mice (OVX) compared to soy isoflavones (SI) was evaluated to assess the efficacy of PFI (Experiment 2). OVX control fed a control diet, OVX fed a PFI diet (the recommended human intake of PFI), OVX fed a PFI20 diet (20- times the recommended PFI), OVX fed an SI diet (the recommended human intake of SI), and OVX fed an SI20 diet (20 -times the recommended intake of SI) for 28 days in Experiment 2. Body, liver, and visceral fat weights were not affected by the PFI, PFI20, SI, or SI20 diets. The hepatic CYP1A and CYP3A activities were elevated by the SI20 treatment. Ovariectomy-induced bone loss was inhibited by the SI20 treatment, but not by the PFI20 treatment. These results suggest that (1) PFI intake in human doses had no oestrogenic properties and did not affect CYP activity in the liver; (2) there was no evidence that PFI affects the amount of visceral fat in OVX mice.

## 1. Introduction

*Pueraria* (kudzu) is a vine that is indigenous to Eastern Asia. It contains puerarin and other functional components and is used in the production of both pharmaceuticals and health foods. In the Japanese Pharmacopoeia, products of kudzu root containing more than 2% puerarin as dry matter from the roots of *Pueraria lobata (Willd.)* Ohwi is used as crude drugs [1]. Kudzu-derived medicines are reported to relieve fever, lessen stiffness and pain, and act as an antiphlogistic [2], and kudzu flower tea is widely consumed in China. Recently, kudzu flower extracts have been used as health foods in Japan, as they are reported to be an effective treatment for hangovers and obesity in Japan [3,4]. The kudzu flower extract from *Pueraria thomsonii* contains isoflavones such as tectoridin, tectorigenin 7-*O*-xylosylglucoside, tectorigenin, 6-hydroxygenistein-6,7-digucoside, glycitin, glycitein, and genistein [5].

In recent years, many functional foods with indicated health claims such as Foods for Specified Health Uses (FOSHU) and Foods with Function Claims (FFC) have become available in Japan [6]. FOSHU are functional foods that have been approved by the Consumer Affairs Agency (CAA) and the physiological effects on the human body of the principal ingredients are labelled [7]. FFC have been released by CAA, which permits the labelling of structure and function claims under the industry’s own responsibility. However, there are many so-called “health foods” other than FOSHU and FFC on the Japanese market, the efficacy and safety of which have not yet been officially evaluated.

The Ministry of Health, Labour and Welfare are responsible for the food category “Borderline of pharmaceuticals and non-pharmaceuticals” in raw materials, and they categorise the root of kudzu as a pharmaceutical [8]. Conversely, the flower of the kudzu is not categorised as a pharmaceutical, and is instead listed as a “natural raw material”. These are not judged as pharmaceuticals, unless they are described with medical claims [8]. For this reason, kudzu flower extract is widely used as a health food ingredient with unproven health benefits. Additionally, in recent years, FFC containing extract of kudzu flower have become widely commercially available as functional foods to reduce visceral fat.

Certain herbal extracts have been reported to modulate the activity of drug-metabolising enzymes, leading to metabolism-mediated herb–drug interactions [9]. The liver is the primary locus of reactions involving cytochrome P450s (CYPs), which plays significant roles in the first-pass metabolism of many orally-administered drugs or xenobiotics [10]. More than 85% of marketed drugs are known to be metabolised by CYPs [11].

Age-related changes in the liver and in the release of sex hormones are reported to influence the expression and activities of CYPs [12]. Thus, herb–drug interactions are important processes, considering that herbal supplements are often prescribed medicines consumed concomitantly in postmenopausal women, whose drug metabolism capacity is affected by ageing and low levels of sex hormones [13,14,15]. Moreover, it has been reported that, in Japan, women consume more health foods than men, and that women over the age of 50 use these foods more frequently than those under the age of 50 [16]. However, few studies have investigated the influence of factors such as age and gender on herb–drug interactions. Thus, the safety profile of kudzu flower extract in postmenopausal patients has not yet been studied in terms of its drug metabolism-mediated effects.

Kudzu flower extract, primarily derived from *Pueraria thomsonii*, contains isoflavones. Isoflavones induce weak oestrogen-like effects and are classified as phytoestrogens [17]. Thus, kudzu flower extract might be used as an alternative to oestrogen-based menopausal therapies by menopausal women, who are likely to take it concomitantly with other medicines. In 2006, the Food Safety Commission of Japan announced their recommendations concerning the consumption of soy isoflavones (SI), and the upper limit of safe daily consumption of SI was set to total 75 mg/day for aglycones and 30 mg/day for supplements [18]. However, while guidelines exist concerning the safe intake of SI, comparatively few studies have investigated the safe intake of isoflavones derived from kudzu flower extracts (PFI).

Therefore, this study evaluated the safety and efficacy of PFI relative to that of SI in ovariectomised (OVX) mice, which have conditions similar to the oestrogen-deprived status of postmenopausal women. We first evaluated the influences of various PFI intakes: normal PFI intake (the recommended human intake of PFI), 20-times the recommended human intake of PFI (PFI20), and 50-times the recommended human intake of PFI (PFI50), on hepatic drug metabolism activity and the indices of liver function in normal female mice for 14 days. Then, we assessed the effects of PFI, PFI20, SI intake (SI in human intake), and 20-times the recommended human intake of SI (SI20) on hepatic drug metabolism activity, the indices of liver function and oestrogenic effects in OVX mice for 28 days.

## 2. Results

### 2.1. Experiment 1: The Effects of PFI on Abdominal Fat and Hepatic CYP Activity in Female Normal Mice for 14 Days

#### 2.1.1. PFI had no Effect on Body and Organ Weights, Plasm Biomarkers of Liver Function in Normal Female Mice

We examined the effects of PFI intake on body weight, liver and uterine weights, the liver function markers to assess the safety, and abdominal fat weight to assess the efficacy in female normal mice. There were no significant effects of PFI, PFI20, or PFI50 on body weight, liver, abdominal fat, and uterine weight for 14 days (Table 1). No PFI treatments at any doses had an effect on the activities of ALP and AST or the concentrations of total cholesterol and triglyceride in the plasma (Table S1).

#### 2.1.2. Excess PFI Intake Up-Regulated Specific Hepatic CYP mRNA Expressions in Female Normal Mice

**Figure 1:**
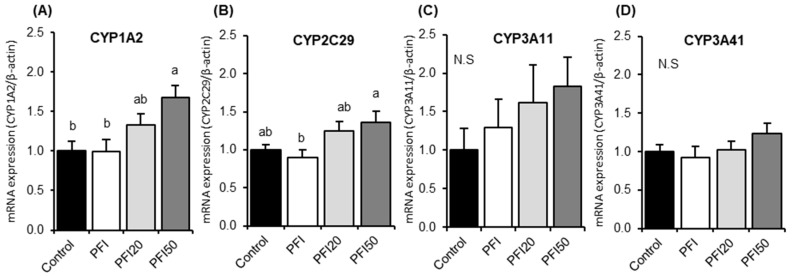
Hepatic mRNA expressions of (**A**) CYP1A2, (**B**) CYP2C29, (**C**) CYP3A11 and (**D**) CYP 3A41 in normal female mice in Experiment 1. Control, mice fed a control diet; PFI, mice fed a *Pueraria* flower (kudzu) isoflavones (PFI) diet (the recommended human intake of PFI); PFI20, mice fed a PFI20 diet (20-times the recommended human intake of PFI); PFI50, mice fed PFI50 diet (50-times the recommended human intake of PFI) for 14 days. Values are the means ± SEMs (*n* = 8). The data were analysed using one-way analysis of variance (ANOVA). Differences between groups were assessed by Tukey’s post hoc test. Differences were considered significant when *p* < 0.05. ^a, b, c^ Mean values with different letters were significantly different.

We examined the effects of PFI intake on hepatic CYP activities and mRNA expression to assess its safety in female normal mice. There were no differences in the activities of CYPs (CYP1A1, CYP1A2, CYP2C9, CYP2D6 and CYP3A4) among all the groups (Figure S1). However, the mRNA expressions of CYP1A2 and CYP2C29 in mice in the PFI50 treatment group were significantly higher than those in the control group and/or the PFI group (Figure 1A,B). There were no significant differences in the mRNA expressions of CYP1A2 and CYP2C29 among the control, PFI, and PFI20 groups. There were no significant differences in the mRNA expression of CYP3A11 and CYP3A41 among all the groups (Figure 1C,D).

### 2.2. Experiment 2: The Effects of PFI and SI on Abdominal Fat, Uterine Weight, Hepatic CYP Activity and Bone Mineral Density (BMD) in OVX Mice for 28 Days

#### 2.2.1. PFI Had no Effect on Body and Organ Weights, Plasm Biomarkers of Liver Function in OVX Mice

We examined the effects of PFI intake on body weight, liver and uterine weights, and the liver function markers to assess its safety, and abdominal fat weight to assess its efficacy in OVX mice. There were no significant effects of the PFI, PFI20, SI, and SI20 treatments on body weight and liver and abdominal fat weights for 28 days (Table 2). Uterine weights from all OVX groups were significantly lower than in the sham group. Uterine weight in the OVX + SI20 group was significantly higher than in the OVX group (*p* = 0.040), and it tended to be higher than those in the OVX + PFI, OVX + PFI 20 and OVX + SI groups (*p* = 0.081, *p* = 0.059, and *p* = 0.053, respectively) (Table 2). The treatments with PFI, PFI20, SI, and SI20 had no effect on the activities of ALP and AST and the concentrations of total cholesterol and triglyceride in the plasma (Table S2).

#### 2.2.2. PFI Had no Effect on Hepatic CYP Activities and mRNA Expression in OVX Mice

**Figure 2:**
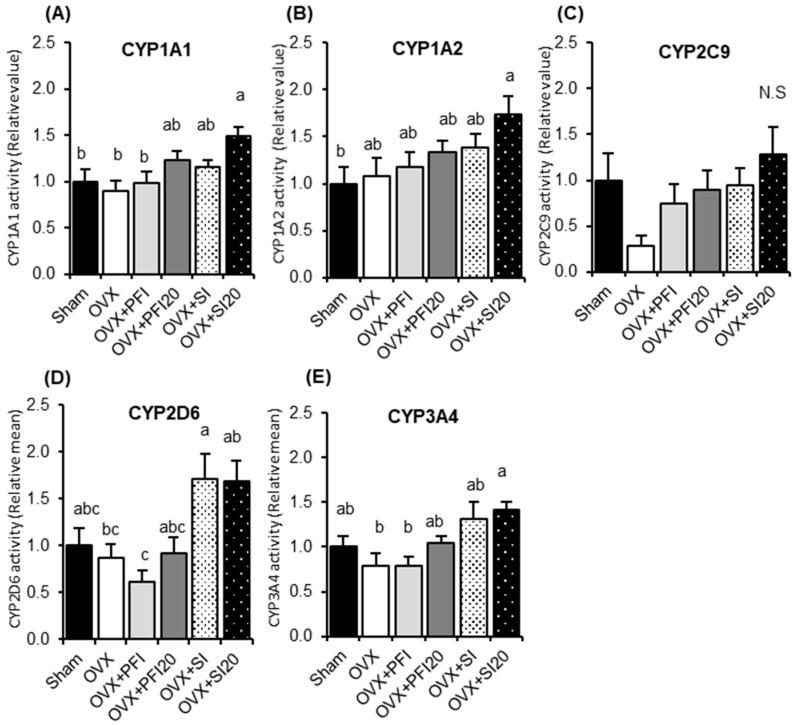
Hepatic activities of (**A**) CYP1A1, (**B**) CYP1A2, (**C**) CYP2C9, (**D**) CYP2D6 and (**E**) CYP3A4 in OVX mice in Experiment 2. Sham, sham-operated mice fed a control diet; OVX, ovariectomised mice (OVX) fed a control diet; OVX + PFI, OVX fed a *Pueraria* flower (kudzu) isoflavones (PFI) diet (the recommended human intake of PFI); OVX + PFI20, OVX fed a PFI20 diet (20-times the recommended human intake of PFI); OVX + SI, OVX fed an soy isoflavones (SI) diet (the recommended human intake of SI); OVX + SI20, OVX fed a SI20 diet (20-times the recommended human intake of SI) for 28 days. Values are the means ± SEMs (*n* = 8). The data were analysed using one-way analysis of variance (ANOVA). Differences between groups were assessed by Tukey’s post hoc test. Differences were considered significant when *p* < 0.05. ^a, b, c^ Mean values with different letters were significantly different.

We examined the effects of PFI intake on hepatic CYP activities and mRNA expression in OVX mice to assess its safety. The hepatic activities of the evaluated CYPs are shown in Figure 2. There were no significant differences in CYP activities between OVX and OVX + PFI groups. CYP1A1 activity was significantly higher in the OVX + SI20 group than those in the Sham, the OVX, and OVX + PFI groups (*p* = 0.035, *p* = 0.006 or *p* = 0.027, respectively) (Figure 2A). CYP 1A2 activity was significantly higher in the OVX + SI20 group than that in the sham group (p = 0.034) and tended to be higher than in the OVX group (*p* = 0.077) (Figure 2B). Although CYP2C9 activity in the OVX + SI and OVX + SI20 groups tended to be higher than in the OVX group (*p* = 0.091 and *p* = 0.099, respectively), there were no significant differences among all the groups (Figure 2C). CYP2D6 activity in the OVX + SI group was significantly higher than in the OVX (*p* = 0.043) and PFI groups (*p* = 0.003) (Figure 2D). CYP2D6 activity in the OVX + SI20 group was significantly higher than in PFI group (*p* = 0.004), and tended to be higher in the OVX (*p* = 0.050) and the OVX + PFI20 (*p* = 0.077) groups. There were no significant differences in CYP2D6 activity among the sham, the OVX, OVX + PFI and OVX + PFI20 groups. CYP3A4 activity in the OVX + SI20 group was significantly higher than in the OVX and the OVX + PFI groups (*p* = 0.023 and *p* = 0.005, respectively), and tended to be higher than in the OVX + PFI20 group (*p* = 0.084) (Figure 2E).

**Figure 3:**
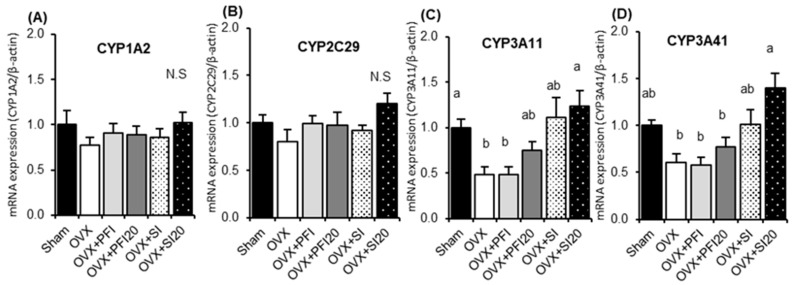
Hepatic mRNA expressions of (**A**) CYP1A2, (**B**) CYP2C29, (**C**) CYP3A11 and (**D**) CYP 3A41 in OVX mice in Experiment 2. Sham, sham-operated mice fed a control diet; OVX, ovariectomised mice (OVX) fed a control diet; OVX + PFI, OVX fed a *Pueraria* flower (kudzu) isoflavones (PFI) diet (the recommended human intake of PFI); OVX + PFI20, OVX fed a PFI20 diet (20-times the recommended human intake of PFI); OVX + SI, OVX fed an soy isoflavones (SI) diet (the recommended human intake of SI); OVX + SI20, OVX fed a SI20 diet (20-times the recommended human intake of SI) for 28 days. Values are the means ± SEMs (*n* = 8). The data were analysed using one-way analysis of variance (ANOVA). Differences between groups were assessed by Tukey’s post hoc test. Differences were considered significant when *p* < 0.05. ^a, b^ Mean values with different letters were significantly different.

PFI or SI treatment had no significant effects on the expression of CYP1A2 and CYP2C29 (Figure 3A,B). OVX induced a significant decrease in the mRNA expression of CYP3A11 (Figure 3C) (*p* = 0.012). The mRNA expression of CYP3A11 and CYP3A41 on the OVX + SI20 group was significantly higher than those in the OVX group (*p* = 0.024 and *p* < 0.001, respectively) (Figure 3C,D). There were no significant effects of PFI on the expression of CYP3A11 and CYP3A41.

#### 2.2.3. PFI Had no Effect on Femoral Bone Mineral Density (BMD) in OVX Mice

**Figure 4:**
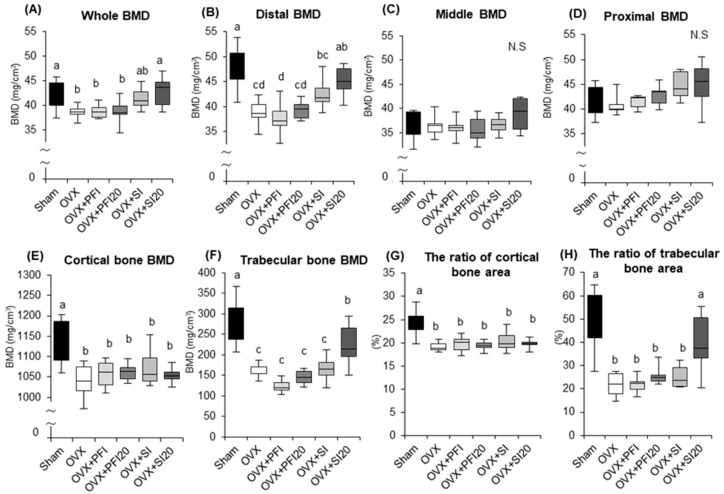
Bone mineral density (BMD) and parameters of the femurs in OVX mice in Experiment 2. (**A**) Whole BMD, (**B**) distal BMD, (**C**) middle BMD and (**D**) proximal BMD in femurs. (**E**) cortical bone BMD, (**F**) trabecular bone BMD, (**G**) the ratio of the cortical bone area and (**H**) the ratio of trabecular bone area of distal femur. Sham, sham-operated mice fed a control diet; OVX, ovariectomised mice (OVX) fed a control diet; OVX + PFI, OVX fed a *Pueraria* flower (kudzu) isoflavones (PFI) diet (the recommended human intake of PFI); OVX + PFI20, OVX fed a PFI20 diet (20-times the recommended human intake of PFI); OVX + SI, OVX fed an soy isoflavones (SI) diet (the recommended human intake of SI); OVX + SI20, OVX fed a SI20 diet (20-times the recommended human intake of SI) for 28 days. Values are the means ± SEMs (*n* = 8). The statistical significance of differences in femoral BMD was determined by analysis of covariance (ANCOVA) and a Bonferroni significant difference test. Body weight was used as a covariate in the analyses of femoral BMD to adjust for possible confounding effects. Differences were considered significant when *p* < 0.05. ^a, b, c, d^ Mean values with different letters were significantly different.

We examined the effects of PFI intake on femoral BMD in OVX mice to assess its efficacy. Whole femoral BMD, as well as the BMD of the distal region of the femur of OVX mice were both significantly lower than in the sham group (Figure 4A,B) (*p* = 0.029, *p* < 0.001, respectively). The SI20 treatment significantly attenuated femoral bone loss and bone loss in the distal region of the femur (relative to the OVX group: *p* = 0.022 and *p* = 0.007, respectively) (Figure 4A,B). There were no significant effects of PFI on whole femoral BMD and the BMD of the distal region of the femur (Figure 4A,B). There were no significant differences in BMDs of the middle and proximal femoral regions among all the groups (Figure 4C,D).

The cortical and trabecular bone BMDs, and the ratios of cortical to trabecular bone area were both significantly lower in the OVX groups than in the sham group (Figure 4E−H). SI20 treatment significantly attenuated trabecular bone BMD loss and significantly decreased the ratio of trabecular bone area (relative to the OVX group: *p* = 0.024 and *p* = 0.001, respectively), but there were no significant effects of PFI on these parameters (Figure 4F,H). There were no significant differences on the cortical bone BMD and the ratio of the cortical bone area among the OVX groups (Figure 3E,G).

## 3. Discussion

This study investigated the safety and potential health benefits of isoflavones derived from the flower of the kudzu. First, we examined the effects of PFI intake on hepatic drug metabolism activity, the indices of liver function in normal female mice for 14 days. Then, OVX mice, which have conditions similar to the oestrogen-deprived status of postmenopausal women, were used to investigate the oestrogenic effects of the PFI and to determine the potential interaction between drugs and PFI by making a 28-day comparison with the effects of SI. We demonstrated, for the first time, that PFI intake at the recommended human dose has no oestrogenic effects and does not affect CYP activity in the liver; moreover, PFI did not affect visceral fat weight in OVX mice. In the first experiment, our results showed that the expression of hepatic CYP1A2 and CYP2C29 in mice treated with PFI at 50-times the recommended human dose was significantly higher than those in control mice (Figure 1A,B). However, there were no observable effects of PFI on the activities of hepatic CYP1A2 and CYP2C9 (Figure S1). Although some studies investigating the interaction between drugs and isoflavone-rich extract (other than tectorigenins) from the Pueraria genus “root” have been performed [19,20,21], no studies have investigated the effects of the extracts of plants from the Pueraria genus “flower” regarding their isoflavones, especially tectorigenins. The identities of the non-isoflavone compounds in PFI used in our study are unclear. Lu et al. reported that the isoflavones and saponin were contained in the samples of herbal materials from the *Pueraria thomsonii* flower extract, which contained 30.4–112.0 mg/g and 9.7–42.4 mg/g of isoflavones and saponin, respectively [22]. Thus, PFI in our study might contain some saponins. A previous study has shown that *Panax notoginseng* saponins treated for 1-week increased CYP1A2 activity in rats [23]. We speculate that the saponin content of PFI, when treated at concentrations 50-times greater than the recommended human dose, might also be responsible for the up-regulation of CYP1A2 expression we observed in our experiment.

On the other hand, based on our results, the increase in CYP1A2 activity did not correspond to an increase in the expression of CYP1A2. Previous reports of the interaction between drugs and herbs and its effects on the expression of hepatic CYPs and their activities were limited, and their expressions did not necessarily reflect their activities [24,25].

In the second experiment, we assessed the effects of PFI, PFI20, SI, and SI20 on hepatic drug metabolism and oestrogenic effects in OVX mice for 28 days. PFI concentrations 50-times greater than the recommended human dose were considered to be too strong to assess both the effects of PFI and oestrogen deficiency in OVX mice. Hence, the effect of a PFI concentration that was 20-times greater than the recommended human dose was examined in OVX mice with regard to the hepatic CYP activity, oestrogenic activity, and visceral fat weight. There were no significant effects of the PFI and PFI20 treatments on hepatic CYP activities and the expressions of CYPs in OVX mice (Figure 2 and Figure 3). The hepatic activities of CYP1A1, CYP1A2, CYP2D6, and CYP3A4 were significantly increased (Figure 2A,B,D,E), and the expressions of CYP3A11 and CYP3A41 were up-regulated in the SI20 treatment in OVX mice (Figure 3C,D). It has been reported that genistein and daidzein, typical SI, partially inhibit CYP3A4 and CYP2C9 activities in the human microsome [26]. In contrast, Xiao et al. reported that genistein resulted in a modest induction of CYP3A in healthy participants [27].

The expression of CYP3A41 has been reported as a female-predominant expression [28]. In our study, the expression of CYP3A41 in OVX mice tended to be lower than in sham mice, but it was significantly increased in SI20-treated mice (Figure 3D). These results suggest that oestrogen deficiency might change the expression of CYP3A4 activity.

The PFI and SI treatments did not affect the liver weight or hepatic function indices such as blood ALT and AST activities in both experiments (Table 1, Table 2, Tables S1 and S2). These results suggest that PFI intake in doses that were less than 20-times the recommended human dose does not result in any strong adverse effects on the liver of female normal mice and OVX mice.

PFI intake at the recommended human doses and doses 20-times greater had no effect on uterine weight (Table 2 and Table 3). Uterine weight in the SI20 treatment group was higher than in OVX mice, but was still lower than in the sham mice (Table 2). Kamiya et al. reported that SI products induced an increase in uterine weight at doses of 500 mg/kg in OVX rats, whereas the kudzu flower extract did not cause it, and the ER-binding affinity of the related compound tectorigenin was approximately 0.02–0.04 that of genistein in vitro [17]. Moreover, our study demonstrated that the femoral BMD and trabecular BMD of the distal femur were higher in OVX mice in the SI20 treatment relative to untreated OVX mice, and that the BMDs of mice in the PFI20 treatment did not exhibit a similar response. Thus, based on these results, PFI intake at doses less than 20-times the recommended human intake does not appear to cause oestrogenic effects.

It has been reported that kudzu (*Pueraria thomsonii*) flower extract decreases visceral fat in overweight humans [3], and a diet containing 5% kudzu (*Pueraria thomsonii*) flower extract suppressed body weight increases, hepatic triglyceride levels, and visceral fat weight compared to high-fat diet-fed control mice [5]. Our results showed that PFI, PFI20, and PFI50 had no effects on abdominal and blood fat levels in normal female mice and OVX mice. Our results are not consistent with those of the previous studies [3,5]. We used OVX mice fed a standard diet and the doses of PFI used (PFI20; PFI 0.93% diet) were lower than in previous studies [5]. These differences in our and other experimental protocols may explain the different results we obtained.

Our results suggest that (1) PFI intake at the recommended human dose has no oestrogenic effects and does not affect CYP activity in the liver upon safety assessment; and (2) PFI did not affect visceral fat weight as determined by the efficacy assessment in a postmenopausal mice model. Thus, we speculate that the physiological effects of PFI are weaker than those of SI. Overall, our study provides valuable information concerning the safety and efficacy of using PFI as an FFC or a botanical supplement in postmenopausal women.

## 4. Materials and Methods

### 4.1. Materials

PFI was kindly provided by Toyoshinyaku Co. Ltd. (Pueraria (kudzu) flower extract, Tosu-shi, Japan). PFI contains 4.7% tectoridin, 8.4% tectorigenin 7-*O*-xylosylglucoside, and 0.83% tectorigenin, and 3.71% other isoflavones (3.38% 6-hydroxygenistein-6,7-digucoside, 0.17% glycitin, 0.10% glycitein, and 0.06% genistein) [5]. The SI extract was purchased from Fujicco Co. Ltd. (Fujiflavone P40, Kobe, Japan). SI extract (isoflavones content, 51.49%; 32.00% aglycone equivalents) is an isoflavone conjugate, with the following aglycones were present in 100 mg of conjugates: 33 mg daidzein, 8.5 mg genistein, and 15 mg glycitein.

### 4.2. Animals, Diets and Experimental Design

Female ddY strain mice, aged 8 weeks, were purchased from the Shizuoka Laboratory Animal Center (Shizuoka, Japan). Mice were housed in individual cages in a temperature- and humidity-controlled room (23 °C ± 1 °C and relative humidity of 60 ± 5%) with a 12-h light–dark cycle. Mice were given free access to an AIN-93G diet and distilled water for 4 days before Experiments 1 and 2 [29]. All procedures involving animals were in accordance with the Guidelines for the care and use of laboratory animals by the National Institute of Biomedical Innovation, Health and Nutrition (Tokyo, Japan) and the ethics committee approved the study protocol (DS27-59R1, 3 April 2017).

#### 4.2.1. Experiment 1: The Effects of PFI on Abdominal Fat and Hepatic CYP Activity in Female Normal Mice for 14 Days

Mice were randomly divided into four groups (all groups, n = 8 each): the control mice (Control), the mice fed a 0.047% PFI-supplemented diet (PFI), mice fed a 0.93% PFI-supplemented diet (PFI20), and the mice fed a 23.3% PFI-supplemented diet (PFI50). Each PFI was mixed into the control powder diet that formed the based on AIN-93G, respectively. The mice were pair-fed their respective diets for 14 days, with free access to distilled water during this period. The amount of food intake was weighted and regulated the PFI intake during the experimental period. One feeding container was used for one mouse. The amount of food intake of each mouse was calculated by subtracting the amount of the remaining diet from the amount of the fed diet. The amount of average food intake in each group was calculated, and the food intake was regulated by matching the feed amount in all groups. The amount of food intake was measured and regulated 4 times a week.

After 14 days of treatment, mice were euthanised by exsanguination under anaesthesia, and then blood was collected in vacutainers and centrifuged at 700× *g* at 4 °C for 15 min. The plasma was removed and stored at −80 °C until assayed. The uterus and abdominal fat were removed and their wet weights were recorded. The liver was removed, then it was washed gently with physiological saline (0.9%) and wiped. One part of the tissue was completely submerged in RNAlater^®^ (Qiagen, Hilden, Germany), another part of the tissue was immediately placed on dry ice and stored at −80 °C until assay.

#### 4.2.2. Experiment 2: The Effects of PFI and SI on Abdominal Fat, Uterine Weight, Hepatic CYP Activity and BMD in OVX Mice for 28 Days

Mice were sham-operated (Sham group, *n* = 8 each) or underwent ovariectomy on the same day. Ovariectomised (OVX) mice were divided into four groups (all groups, *n* = 8 each): the OVX control mice (OVX), the OVX mice fed a 0.047% PFI-supplemented diet (OVX + PFI), the OVX mice fed a 0.94% PFI-supplemented diet (OVX + PFI20), the OVX mice fed a 0.013% SI-supplemented diet (OVX + SI), and the OVX mice fed a 0.26% SI-supplemented diet (OVX + SI20). Each PFI was mixed into the control powder diet based on AIN-93G, respectively. The mice were pair-fed their respective diets for 28 days, with free access to distilled water during this period. The amount of food intake was weighed and regulated the PFI intake same as experiment 1. After 28 days of treatment, mice were euthanised by exsanguination under anaesthesia, and then blood was treated by the same method as Experiment 1. The uterus and abdominal fat were removed and their wet weights were recorded. The liver was removed, then it was treated using the same methods as Experiment 1. The left femur was removed, submerged in 70% ethanol, and stored at 4 °C until measurement of the BMD was performed.

Table 3 shows the composition of the experimental diets, which were prepared according to the AIN-93G formulation [29]. The diets used in these experiments were AIN-93G, containing 0.047%, 0.94%, or 2.35% PFI. The recommended human daily dose of PFI for humans though FFC in Japan is 42 mg/day as the isoflavones from the group of tectorigenins (approximately 0.80 mg/kg body weight (BW)). This dose translated to 10 mg/kg BW of PFI, for mice, using the body surface area normalization method [30]. Because mice (30 g BW) consume approximately 4.5 g of diet/day, 0.047% in the diet is similar to a 10 mg/kg BW intake. Therefore, we examined the effect of 0.047%, 0.94% and 2.35% PFI in these diets; these doses were the recommended human intake and, its 10-times and 50-times the recommended human intake, respectively. The SI and SI20 diets contained 0.019% and 0.39% SI, where the dose corresponded to the content of the group of isoflavones from tectorigenins in the PFI and PFI20 diets.

### 4.3. Preparation of the Liver Microsomal Fraction

Livers were homogenized in 50 mM Tris-HCl buffer, containing 0.25 M sucrose (pH 7.4) with a polytron homogenizer. The homogenate was centrifuged at 10,000× *g* for 30 min at 4 °C, and the supernatant was collected. The supernatant was centrifuged again at 105,000× *g* for 60 min at 4 °C, and the supernatant was discarded. The pellet was resuspended in 50 mM Tris-HCl buffer (pH 7.4) and used as the liver microsomal fraction. Protein concentrations were determined using the BCA protein assay kit (Pierce, Rockford, IL, USA).

### 4.4. Measurement of CYP Activity

The activity of each CYP subtype in the liver microsomal fraction was measured using a luminescence method with the P450-Glo^TM^ CYP1A1 System (Luciferin-CEE) assay, CYP1A2 System (Luciferin-ME) Assay, CYP2C9 System (Luciferin-H) Assay, CYP2D6 System (Luciferin-ME EGE) Assay, CYP 3A4 system (Luciferin-PPXE) Assay, and Detection System (Promega Co., Madison, WI, USA). CYP activity was adjusted to the protein concentration, and the results were represented as a percentage of the control or the sham groups.

### 4.5. RNA Extraction from Liver and Quantitative Real-Time PCR

Total RNA was extracted from the liver using the RiboPure™ RNA Purification Kit (Ambion by Life Technologies, Carlsbad, CA, USA), according to manufacturer’s instructions. Complementary DNA (cDNA) was synthesized from 1 μg of total RNA, using PrimeScript RT Master Mix (TaKaRa Bio, Shiga, Japan). cDNA was quantified by real-time PCR using SYBR Premix Ex Taq II (Takara Bio, Shiga, Japan). The PCR conditions were 95 °C for 30 s, followed by 40 cycles of 95 °C for 5 s and 60 °C for 30 s. The primer sequences are shown in Table S3. The relative gene expression from each liver sample of target genes compared with the β-actin reference gene was determined using the comparative Ct threshold method and the DDCt was used for relative quantification. The results are expressed as the fold-change relative to the control or the sham groups.

### 4.6. Markers of Liver Function and the Concentrations of Total cholesterol and Triglyceride in Plasma

AST and ALT activities, which are markers of liver function, in plasma were determined using enzymic methods. The concentrations of total cholesterol and triglyceride in plasma were measured by the enzymic method. These analyses were conducted by Oriental yeast Co., Ltd. (Tokyo, Japan).

### 4.7. Radiographic Analyses of the Femur

The BMD of the femur was quantified by dual-energy X-ray absorptiometry (DEXA, DCS-600EX-RIII; Aloka, Tokyo, Japan) and calculated using the bone mineral content of the measured area. The scanned area of the mouse femur was divided into three equal parts: proximal, midshaft, and distal.

### 4.8. Microcomputed Tomography (μCT) Analysis of the Femur

Distal femurs were scanned at 48 μm intervals using an experimental animal CT system (LaTheta LCT-200; Hitachi Aloka Medical, Tokyo, Japan). Analyses of distal femurs were performed in a region of the trabecular and cortical bone to the growth plate extending 1.4 mm towards the diaphysis.

### 4.9. Statistical Analyses

Data are presented as the mean ± standard error of the mean (SEM). The data were analysed using one-way analysis of variance (ANOVA). Differences among the groups were assessed by Tukey’s post hoc test. If the data did not have a normal distribution and their variances were not equivalent, a Games-Howell non-parametric multiple comparison test was carried out to determine significant differences between groups. The statistical significance of differences in femoral BMD was determined by analysis of covariance (ANCOVA) and Bonferroni significant difference test. Body weight was used as a covariate in the analyses of femoral BMD to adjust for possible confounding effects. Differences were considered significant if *p* < 0.05. Statistical analyses were conducted using SPSS Statistics, version 19 (IBM, Armonk, NY, USA).

## Figures and Tables

**Table 1:** Body weight, food intake, and wet weight of liver, abdominal fat, and uterine in mice in Experiment 1.

	Control	PFI	PFI 20	PFI 50	ANOVA *p* Value
Body weight					
Initial body weight (g)	28.3 ± 0.2	28.3 ± 0.2	28.3 ± 0.2	28.3 ± 0.2	1.000
Final body weight (g)	31.5 ± 1.1	33.4 ± 0.6	32.6 ± 0.7	32.1 ± 0.9	0.454
Total food intake (g)	60.8 ± 0.3	60.7 ± 0.2	61.0 ± 0.0	60.5 ± 0.6	0.709
Organ weights					
Liver (mg/10g BW *)	473.4 ± 25.1	475.3 ± 25.5	477.2 ± 17.7	485.0 ± 16.5	0.982
Abdominal fat (mg/10g BW)	502.6 ± 57.8	585.0 ± 32.8	600.5 ± 71.1	532.8 ± 43.0	0.542
Uterine (mg/10g BW)	47.9 ± 6.8	52.4 ± 6.7	42.0 ± 5.6	41.2 ± 4.0	0.503

Control, mice fed a control diet; PFI, mice fed a *Pueraria* flower (kudzu) isoflavones (PFI) diet (the recommended human intake of PFI); PFI20 mice fed a PFI20 diet (20-times the recommended human intake of PFI); PFI50, mice fed PFI50 diet (50-times the recommended human intake of PFI) for 14 days. Values are the means ± SEMs (*n* = 8). The data were analysed using one-way analysis of variance (ANOVA). Differences between groups were assessed by Tukey’s post hoc test. Differences were considered significant when *p* < 0.05. * BW: body weight.

**Table 2:** Body weight, food intake, and wet weight of liver, abdominal fat, and uterine in mice in Experiment 2.

	Sham	OVX	OVX + PFI	OVX + PFI 20	OVX + SI	OVX + SI 20	ANOVA *p* Value
Body weight							
Initial body weight (g)	28.2 ± 0.4	28.0 ± 0.4	28.7 ± 0.5	28.1 ± 0.4	28.2 ± 0.3	28.2 ± 0.4	0.891
Final body weight (g)	35.4 ± 0.5	37.1 ± 1.2	37.6 ± 1.3	38.7 ± 1.1	37.9 ± 1.0	38.5 ± 0.3	0.209
Total food intake (g)	117.3 ± 1.4	117.9 ± 2.4	116.3 ± 2.3	117.8 ± 2.4	118.7 ± 2.0	119.7 ± 1.1	0.884
Organ weights							
Liver (mg/10g BW *)	389.7 ± 10.0	417.4 ± 16.1	405.8 ± 13.1	414.2 ± 22.0	413.8 ± 13.5	429.3 ± 21.2	0.529
Abdominal fat (mg/10g BW)	620.4 ± 77.5	540.4 ± 44.0	491.0 ± 47.2	523.2 ± 55.9	504.8 ± 52.3	507.3 ± 38.3	0.389
Uterine (mg/10g BW)	53.1 ± 12.1 ^a^	6.0 ± 0.4 ^c^	6.4 ± 0.8 ^bc^	6.1 ± 0.7 ^bc^	6.2 ± 0.5 ^bc^	10.7 ± 1.2 ^b^	<0.001

Sham, sham-operated mice fed a control diet; OVX, ovariectomised mice (OVX) fed a control diet; OVX + PFI, OVX fed a *Pueraria* flower (kudzu) isoflavones (PFI) diet (recommended human intake level of PFI); OVX + PFI20, OVX fed a PFI20 diet (20-times the recommended human intake of PFI); OVX + SI, OVX fed an soy isoflavones (SI) diet (the recommended human intake of SI); OVX + SI20, OVX fed a SI20 diet (20-times the recommended human intake of SI) for 28 days. Values are the means ± SEMs (*n* = 8). The data were analysed using one-way analysis of variance (ANOVA). Differences between groups were assessed by Tukey’s post hoc test. Differences were considered significant when *p* < 0.05. ^a, b, c^ Mean values with different letters were significantly different. * BW: body weight.

**Table 3:** Composition of the experimental diets (g/kg diet) ^a^.

Ingredient	Control ^b^	PFI Diet ^c^	PFI 20 Diet ^d^	PFI 50 Diet ^e^	SI Diet ^f^	SI 20 Diet ^g^
Cornstarch	529.5	529.0	520.2	506.2	529.4	526.9
Casein	200	200	200	200	200	200
Sucrose	100	100	100	100	100	100
Corn oil	70	70	70	70	70	70
Cellulose	50	50	50	50	50	50
Mineral mixture ^a^	35	35	35	35	35	35
Vitamin mixture ^a^	10	10	10	10	10	10
L-Cystine	3	3	3	3	3	3
Choline bitartrate	2.5	2.5	2.5	2.5	2.5	2.5
Tert-Butylhydroquinone	0.014	0.014	0.014	0.014	0.014	0.014
*Pueraria* flower isoflavones extract ^h^	-	0.466	9.320	23.300	-	-
Soy isoflavones extract ^i^	-				0.130	2.600
Total	1000	1000	1000	1000	1000	1000

^a^ Prepared according to the AIN-93G formulation [29]. ^b^ Control diet. ^C^ *Pueraria* flower isoflavones extract (PFI) diet. The dose, 0.046% PFI in the diet, was the recommended human intake. ^d^ PFI20 diet. The dose, 0.932% PFI in the diet, was 20-times the recommended human intake. ^e^ PFI50 diet. The dose, 2.33% PFI in the diet, was 50-times the recommended human intake. ^f^ Soy isoflavones extract (SI)-supplement diet. The dose, 0.013% SI in the diet, was the recommended human intake. ^g^ SI20 diet. The dose, 0.26% SI in the diet, was 20-times the recommended human intake. ^h^ PFI (Toyoshinyaku, Tosu-shi, Japan) contained 4.7% tectoridin, 8.4% tectorigenin 7-*O*-xylosylglucoside, and 0.83% tectorigenin, and 3.71% other isoflavones (3.38% 6-hydroxygenistein-6,7-digucoside, 0.17% glycitin, 0.10% glycitein, and 0.06% genistein) [5]. ^i^ SI (Fujiflavone P40^TM^; Fujicco, Kobe Japan) contained 51.49% isoflavones conjugates (32.00% aglycone equivalent), with the following aglycones in 100 mg of conjugates: 33 mg daidzein, 8.5 mg genistein, and 15mg glycitein.

## Supplementary Materials

Supplementary materials can be found at https://www.mdpi.com/1422-0067/20/12/2867/s1. Figure S1, CYP activity in the liver of the mice in Experiment 1.; Table S1, Plasma activities of aspartate aminotransferase, alanine aminotransferase, and concentrations of total cholesterol and triglyceride in mice in Experiment 1.; Table S2, Plasma activities of aspartate aminotransferase, alanine aminotransferase, and concentrations of total cholesterol and triglyceride in mice in Experiment 2.: Table S3, Sequence of primers used for quantitative real-time PCR.

## Abbreviations

ALT	Alanine amino transferase
ANCOVA	Covariate in the analyses
ANOVA	Analysis of variance
AST	Aspartate amino transferase
BMD	Bone mineral density
CAA	The Consumer Affairs Agency
CYP	Cytochrome P-450
FFC	Foods with Function Claims
FOSHU	Foods for Specified Health Uses
OVX	Ovariectomised
PFI	Flower extract of *Pueraria*
SEM	Standard error of the mean
SI	Soy isoflavones
