# Supplementary material for: Safety and Efficacy Assessment of Isoflavones from Pueraria (Kudzu) Flower Extract in Ovariectomised Mice: A Comparison with Soy Isoflavones
Source: Int J Mol Sci. 2019 Jun 12;20(12):2867. doi: 10.3390/ijms20122867 (PMC6627882; doi:10.3390/ijms20122867)
Supplement: Supplementary file 1 [file ijms-20-02867-s001.zip › èïé╠ë╘ÿ_ò╢_final proofed_Supplement Tables and Figure/Tables S1-3_R2_Proof.docx]

**Table S1.** Plasma activities of aspartate aminotransferase, alanine aminotransferase, and concentrations of total cholesterol and triglyceride in mice in Experiment 1.

|  | Control | PFI | PFI 20 | PFI 50 | ANOVA  *p* value |
| --- | --- | --- | --- | --- | --- |
| AST* (IU^$^/L) | 145.3 ± 15.7 | 146.9 ± 12.2 | 132.0 ± 17.2 | 107.6 ± 9.4 | 0.208 |
| ALP^#^ (IU/L) | 23.1 ± 2.7 | 25.6 ± 2.7 | 20.1 ± 1.2 | 22.6 ± 3.4 | 0.566 |
| Total cholesterol (mg/dL) | 115.6 ± 6.3 | 110.3 ± 6.3 | 137.1 ± 13.0 | 129.7 ± 5.1 | 0.111 |
| Triglyceride (mg/dL) | 31.8 ± 7.6 | 36.9 ± 6.5 | 38.0 ± 6.6 | 40.7 ± 4.4 | 0.806 |

Control, mice fed a control diet; PFI, mice fed a *Pueraria* flower (kudzu) isoflavones (PFI) diet (the recommended human intake of PFI); PFI20, mice fed a PFI20 diet (20-times the recommended human intake of PFI); and PFI 50, mice fed PFI50 diet (50-times the recommended human intake of PFI) for 14 days. Values are the means ± SEMs (n = 8). The data were analysed using one-way analysis of variance (ANOVA). Differences between groups were assessed by Tukey’s post hoc test. Differences were considered significant when *p* < 0.05. * AST: Aspartate amino transferase, ^#^ALP: Alanine amino transferase, ^$^ IU: International unit

**Table S2.** Plasma activities of aspartate aminotransferase, alanine aminotransferase, and concentrations of total cholesterol and triglyceride in mice in Experiment 2.

|  | Sham | OVX | OVX +  PFI | OVX +  PFI 20 | OVX +  SI | OVX +  SI 20 | ANOVA  *p* value |
| --- | --- | --- | --- | --- | --- | --- | --- |
| AST* (IU^$^/L) | 175.8 ± 23.7 | 152.3 ± 13.1 | 123.6 ± 16.9 | 130.6 ± 9.2 | 164.6 ± 33.3 | 132.7 ± 22.0 | 0.465 |
| ALP^#^ (IU/L) | 22.7 ± 3.0 | 26.1 ± 4.8 | 16.7 ± 1.2 | 19.3 ± 1.9 | 22.2 ± 2.6 | 21.3 ± 2.9 | 0.560 |
| Total cholesterol (mg/dL) | 136.0 ± 9.7 | 131.4 ± 9.6 | 148.4 ± 6.6 | 140.6 ± 8.2 | 125.0 ± 6.3 | 140.0 ± 8.0 | 0.489 |
| Triglyceride (mg/dL) | 16.8 ± 1.5 | 33.3 ± 9.1 | 25.6 ± 3.3 | 20.3 ± 1.7 | 22.0 ± 5.2 | 25.9 ± 4.8 | 0.864 |

Sham, sham-operated mice fed a control diet; OVX, ovariectomized mice (OVX) fed a control diet; OVX + PFI, OVX fed a *Pueraria* flower (kudzu) isoflavones (PFI) diet (recommended human intake level of PFI); OVX + PFI20, OVX mice fed a PFI20 diet (20-times the recommended human intake of PFI); OVX + SI, OVX fed an soy isoflavones (SI) diet (the recommended human intake of SI); OVX + SI20, OVX fed a SI20 diet (20-times the recommended human intake of SI) for 28 days. Values are the means ± SEMs (n = 8). The data were analysed using one-way analysis of variance (ANOVA). Differences between groups were assessed by Tukey’s post hoc test. Differences were considered significant when *p* < 0.05. ^a, b, c^ Mean values with different letters were significantly different. * AST: Aspartate amino transferase, ^#^ALP: Alanine amino transferase, ^$^ IU: International unit

**Table S3.** Sequence of primers used for quantitative real-time PCR.

|  | Protein (gene) | Forward primer (5’ to 3’) | Reverse primer (5’ to 3’) |
| --- | --- | --- | --- |
|  | β-actin (*Actb*) | 5′-CCACAGCTGAGAGGGAAATC-3′ | 5′-AAGGAAGGCTGGAAAAGAGC-3’ |
|  | CYP*1A2 | 5′-ACAGCAAGGACTTTGTGGAGAA-3′ | 5′-GTGATGTCTTGGATACTGTTCTTGT-3′ |
|  | CYP2C29 | 5′-TGTCACAGCTAAAGTCCAGG-3′ | 5′-CTAGTGGGGAGGAGGTCGAT-3′ |
|  | CYP3A11 | 5′-CTCAATGGTGTGTATATCCCC-3′ | 5′-CCGATGTTCTTAGACACTGCC-3′ |
|  | CYP3A41 | 5′-CTCTACCGATATGGGACCCG-3′ | 5′-GCACAGTGCCTAAAAATGGCA-3′ |

*CYP: cytochrome P-450
