# Supplementary figures and images for: Safety and Efficacy Assessment of Isoflavones from Pueraria (Kudzu) Flower Extract in Ovariectomised Mice: A Comparison with Soy Isoflavones
Source: Int J Mol Sci. 2019 Jun 12;20(12):2867. doi: 10.3390/ijms20122867 (PMC6627882; doi:10.3390/ijms20122867)

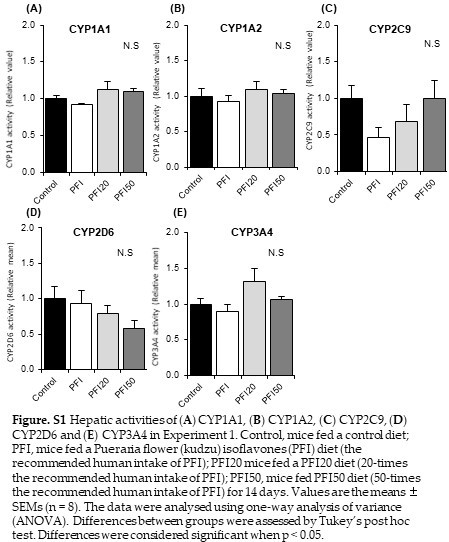

Supplement: Supplementary file 1 [file ijms-20-02867-s001.zip › èïé╠ë╘ÿ_ò╢_final proofed_Supplement Tables and Figure/èïé╠ë╘ùRùêâCâ/âtâëâ{âô_ÿ_ò╢ùp_20190611_JPIGùpfinal.jpg]
